# Lycopene Ameliorates Transplant Arteriosclerosis in Vascular Allograft Transplantation by Regulating the NO/cGMP Pathways and Rho-Associated Kinases Expression

**DOI:** 10.1155/2016/3128280

**Published:** 2016-12-05

**Authors:** Yunqiang He, Peng Xia, Hao Jin, Yan Zhang, Bicheng Chen, Ziqiang Xu

**Affiliations:** ^1^Zhejiang Provincial Top Key Discipline in Surgery, Wenzhou Key Laboratory of Surgery, Department of Surgery, The First Affiliated Hospital of Wenzhou Medical University, Wenzhou, Zhejiang Province 325000, China; ^2^Department of Transplantation, The First Affiliated Hospital of Wenzhou Medical University, Wenzhou, Zhejiang Province 325000, China

## Abstract

*Objective.* Transplant arteriosclerosis is considered one of the major factors affecting the survival time of grafts after organ transplantation. In this study, we proposed a hypothesis of whether lycopene can protect grafted vessels through regulating key proteins expression involved in arteriosclerosis.* Methods.* Allogeneic aortic transplantation was performed using Brow-Norway rats as donors and Lewis rats as recipients. After transplantation, the recipients were divided into two groups: the allograft group and the lycopene group. Negative control rats (isograft group) were also established. Histopathological staining was performed to observe the pathological changes, and the expression levels of Ki-67, caspase-3, Rho-associated kinases, intercellular adhesion molecules (ICAM-1), and eNOS were assessed. Western blotting analysis and real-time PCR were also performed for quantitative analysis.* Results.* The histopathological staining showed that vascular stenosis and intimal thickening were not evident after lycopene treatment. The Ki-67, ROCK1, ROCK2, and ICAM-1 expression levels were significantly decreased. However, eNOS expression in grafted arteries and plasma cGMP concentration were increased after lycopene treatment.* Conclusions.* Lycopene could alleviate vascular arteriosclerosis in allograft transplantation via downregulating Rho-associated kinases and regulating key factor expression through the NO/cGMP pathways, which may provide a potentially effective method for transplant arteriosclerosis in clinical organ transplantation.

## 1. Introduction

Although significant advances in the application of immunosuppressants have significantly increased the success rate of organ transplantation [1], transplant arteriosclerosis (TA) is also considered as one of the major causes of graft failure in a number of transplant patients [2]. Vascular injuries mainly include ischemia-reperfusion injury, the long-term use of immunosuppressive drugs, infiltration of inflammatory cytokines, and acute or chronic graft rejections. In the early stage, these injuries can damage the vascular endothelium, impair the vascular endothelial function, and promote the migration and proliferation of vascular smooth muscle cell (VSMC), which can ultimately lead to arteriosclerosis occlusion [3]. Therefore, vessel protection in grafts has been considered crucial treatment after organ transplantation, which will effectively prevent the occurrence of transplant arteriosclerosis [4].

Lycopene is a natural carotenoid that exists in tomatoes and tomato products. It is considered as one of the most effective singlet oxygen species in carotenoids [5]. Its inactivation capacity is twice that of beta carotene and 100 times that of vitamin E. The study has reported that lycopene can increase the activity and expression level of nitric oxide (NO) [6]. Furthermore, cGMP-dependent protein kinase-I (PKG-I) is considered a critical mediator of the NO/cGMP pathways [7]. It is widely known that lycopene can maintain the normal function of the vascular endothelium by regulating relevant factors expression through the NO/cGMP pathways. Several studies have found that lycopene has an inhibitory effect on thickening of the intima and media of people who smoke. In addition, lycopene can inhibit the adhesion of inflammatory factors in the vascular intima, reducing damage to the vascular intima [8]. Furthermore, activation of Rho-associated kinases (ROCKs) also plays an important role in vascular arteriosclerosis [9]. Therefore, the detection of ROCKs expression levels was performed in this study to demonstrate the mechanisms involved in the progression of transplant arteriosclerosis.

The aim of this study was to investigate the protective effects of lycopene on grafted vessels. A hypothesis was also proposed: whether lycopene protects grafted vessels after vasotransplantation through regulating the NO/cGMP pathways and Rho-associated kinases expression involved in arteriosclerosis. The results demonstrated that lycopene could alleviate the vascular sclerosis of the transplanted arteries, regulate the expression of key factors in relevant signaling pathways of the blood vessels, and reduce the infiltration of inflammatory factors through its antioxidant effects. This may provide a potentially effective treatment approach for transplant arteriosclerosis in clinical organ transplantation.

## 2. Materials and Methods

### 2.1. Animals

Sixteen male Brow-Norway (BN) rats and 32 male Lewis rats at 8 weeks of age (200–220 g) were provided by Vital River Laboratory Animal Technology Co., Ltd. (Beijing, China). All rats were housed with a 12-hour light and 12-hour dark cycle at 24°C ± 1°C, and they were fed ad libitum for a week before the experiments started. All animal procedures were based on the international guidelines and were approved by the Wenzhou Medical University Animal Policy and Welfare Committee.

### 2.2. Aorta Transplantation

Aorta transplantation was performed based on the previously described methods [10]. Syngeneic aortic transplantation was performed in Lewis rats, and allogeneic aortic transplantation was performed using Brow-Norway (BN) rats as donors and Lewis rats as recipients. These rats were anesthetized by intraperitoneal injection of pentobarbital (60 mg/kg). The abdominal aorta (10–15 mm) was harvested from donors after intravenous heparin injection. After sufficient perfusion with saline, the aortic grafts were transplanted into recipients and anastomosed end to end with the abdominal aorta using noninterrupted 10-0 nylon sutures. The total operation ischemic time was consistently limited to 30 minutes. The operation was considered successful when there was complete patency of the grafted arteries without significant anastomotic bleeding. Then, the recipients were divided into 3 groups. The first group (*n* = 8) was treated for 8 weeks with saline vehicle after an isograft (1 ml/day, isograft group); the second group (*n* = 8) was treated for 8 weeks with saline vehicle after an allograft (allograft group); the third group (*n* = 8) was treated for 8 weeks with lycopene (30 mg/kg/d, lycopene group) after an allograft. After the operations, all recipients were treated with ceftazidime (100 mg/kg) for 3 consecutive days.

### 2.3. Histopathology

All grafts were harvested 8 weeks after transplantation. Graft vessel tissues were fixed overnight in 4% paraformaldehyde for histopathological staining. After gradient dehydration in ethanol and xylene, the vessel tissues were embedded in paraffin and cut into 5 *μ*m sections by a microtome. After deparaffinization and rehydration, the sections were stained with hematoxylin and eosin (HE staining) for general morphological examination and pathological analysis. The intima area was calculated by subtracting the lumen area from the area enclosed by the internal elastic lamina lining. The degrees of neointimal hyperplasia and arterial stenosis were measured under the optical microscope (original magnification ×200) and analysed by Image-Pro Plus 6.0 image analysis software (Media Cybernetics, Silver Spring, MD). More than 10 fields were evaluated from each section in each group and the mean value was used. All the measurements were performed on blinded slides.

### 2.4. Enzyme-Linked Immunosorbent Assay (ELISA)

Blood samples were collected 8 weeks after transplantation in each group. Rats' plasma cGMP concentrations were quantitatively measured by using an ELISA kit (R&D Systems, Inc., Minnesota, USA) according to the manufacturer's instructions described previously [11]. All the ELISA experiments were repeated at least three times.

### 2.5. Real-Time Quantitative Reverse Transcription-Polymerase Chain Reaction (Real-Time RT-PCR)

According to the protocol that was previously described by Lin et al. [12], total RNA was extracted from the vessel specimens using TRIzol reagent. The primer sequences were as follows: ROCK1, forward 5′-TGGTTGGGACGTACAGTAAAA-3′ and reverse 5′-GTAAGGAAGGCACAAATGAGA-3′; ROCK2, forward 5′-AATGGGTTAGTCGGTTGG-3′ and reverse 5′-CTTGGTTTGTTTGGAGCA-3′; eNOS, forward 5′-GCCAAACAGGCCTGGCGCAA-3′ and reverse 5′-GTGCTGTCCTGCAGTCCCGA-3′; PKG-I, forward 5′-CTTGGAGTGGGAGGTTTC-3′ and reverse 5′-AATGTGTCGTTTCTTGAGG-3′; ICAM-1, forward 5′-TATCCATCCATCCCACAG-3′ and reverse 5′-GTTCGTCTTTCATCCAGTTAGT-3′; *β*-actin, forward 5′-TACAACTCCTTGCAGCTCC-3′ and reverse 5′-ATCTTCATGAGGTAGTCAGTC-3′. The ROCK1, ROCK2, eNOS, PKG-I, and ICAM-1 levels were normalized to that of *β*-actin. The gene expression was determined by real-time quantitative monitoring of PCR reactions with LightCycler System (Roche, Indianapolis, IN, USA). All PCR experiments were performed in at least three independent treatments.

### 2.6. Immunohistochemistry

The paraffin tissue sections were dewaxed in xylene and rehydrated in gradient alcohol. Then, the sections were washed with distilled water, and endogenous peroxidase was blocked by 3% hydrogen peroxide. The heat-mediated antigen retrieval was performed with citrate buffer at pH 6 before starting the IHC staining protocol. Then, the sections were blocked for 60 min with 5% goat sera. Indirect immunoperoxidase staining of tissue sections was performed, and the samples were incubated overnight with primary antibodies against Ki-67, ICAM-1, (Cambridge, MA, USA), eNOS, activated caspase-3 (Sigma-Aldrich, USA), and ROCK1 and ROCK2 (Proteintech, USA) at 4°C. Then, the tissue sections were washed with phosphate-buffered saline (PBS, 0.01 M), incubated with secondary antibodies, and visualized with diaminobenzidine (DAB, brown color, ZSGB-BIO, Beijing, China) and hematoxylin counterstaining under a microscope. The intensity of positive staining was measured by IOD/area with Image-Pro Plus 6.0 image analysis software (Media Cybernetics, Silver Spring, MD).

### 2.7. Western Blot Analysis

The protein expression of PKG-I was examined by Western blot assays. Briefly, vessel tissues were homogenized in lysis buffer, and the supernatants were collected by centrifugation at 12000 rpm for 15 min at 4°C. After determining the total protein concentration, 50 micrograms of protein per specimen was run on 10% SDS-polyacrylamide gel electrophoresis and transferred to nitrocellulose membranes. After blocking with 5% skim milk for 1 h at room temperature, the membranes were incubated with primary antibodies at 4°C overnight. Then, the membranes were washed with Tris-buffered saline containing 0.05% Tween 20, and they were incubated with secondary horseradish peroxidase-conjugated antibody for 1 h at room temperature. Antigen-antibody complexes were then visualized using an enhanced chemiluminescence kit (Amersham, USA), and the intensity of the protein bands was quantified with Quantity One software (version 4.6.2, Bio-Rad, USA). Rabbit polyclonal antibody PKG-I and mouse monoclonal antibody *β*-actin were purchased from Abcam (Cambridge, MA, USA).

### 2.8. Statistical Analysis

SPSS software (version 19.0; SPSS, Chicago, IL, USA) was used for statistical analyses. All statistical data are presented as the means ± standard error of the mean (SEM). Statistical analyses were performed using one-way analysis of variance. The differences between groups were considered significant at *P* < 0.05.

## 3. Results

### 3.1. Model Building

The aortic transplantation model was considered successfully established when there was complete patency of the grafted arteries without significant anastomotic bleeding 3 days after transplantation. The mean operative duration of surgical vascular transplantation was 41.5 ± 7.5 min. The ischemic time was consistently less than 30 minutes. An abdominal longitudinal incision was performed to observe the grafted vessels 3 days after surgery. In this study, the surgical success percentage was 87.5% (21/24). Eight weeks after transplantation, the blood flow at both the proximal and the distal anastomotic sites was observed. The patency of all successful grafted vessels was adequate.

### 3.2. Lycopene Ameliorated Allograft Arteriosclerosis and VSMCs Proliferation

**Figure 1:**
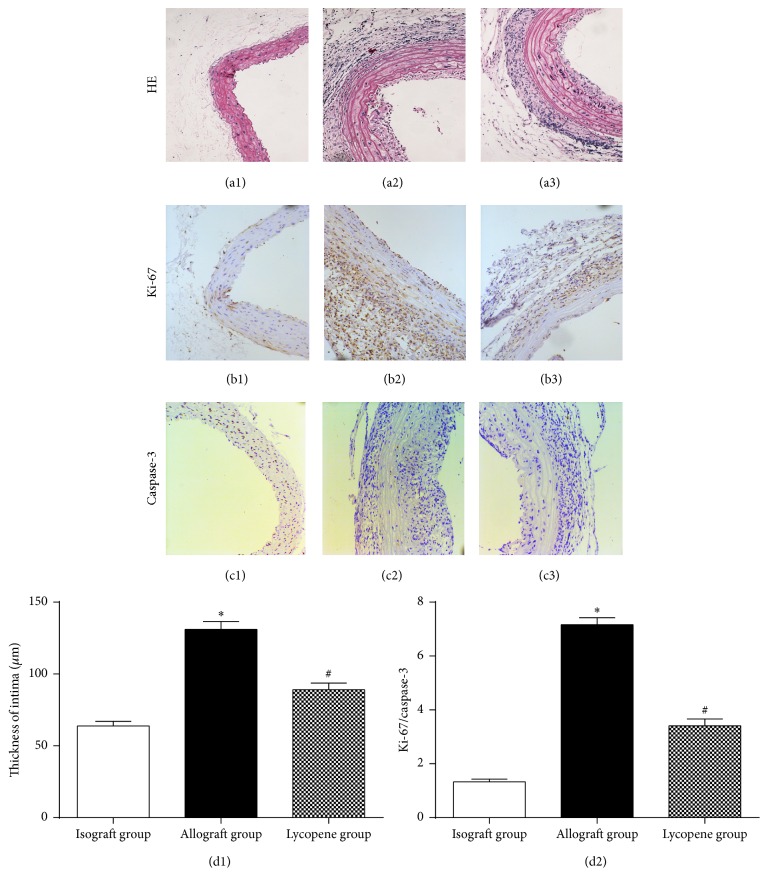
Histopathological staining of grafted vessels and analysis of cell proliferation and apoptosis under light microscopy (original magnification ×200). (a1)–(a3) Hematoxylin-eosin (HE) staining of grafted aortic specimens obtained 8 weeks after transplantation. (b1)–(b3) Immunohistochemical staining of Ki-67 in arterial grafts. (c1)–(c3) Immunohistochemical staining of caspase-3 in grafted arteries. (d1) Thickness of intima in each group (*μ*m, original magnification ×200), ^*∗*^ *P* < 0.001, allograft group compared with the isograft group; ^#^ *P* = 0.017, lycopene group compared with the allograft group. (d2) The positive cells ratio of Ki-67/caspase-3 in each group, ^*∗*^ *P* < 0.001, ^#^ *P* < 0.001. ((a1), (b1), and (c1)) Isograft group; ((a2), (b2), and (c2)) allograft group; ((a3), (b3), and (c3)) lycopene group.

Lycopene could remarkably alleviate neointimal hyperplasia and arteriosclerosis of the aortic grafts. As shown in Figures 1(a1)–1(a3), the HE staining of grafted vessels in each group showed that vascular stenosis and intimal thickening were significant in the allograft group (Figure 1(a2)) compared with the isograft group (a1). This pathological staining also revealed that the inflammatory cells accumulation and leukocyte infiltration were evident in the allograft group. We also measured the degree of vessel stenosis in each group and found that it was more significant in the allograft group. However, these features were not obvious in the lycopene group (Figure 1(a3)). The thickness of the intima in the lycopene group (89.09 ± 4.13 *μ*m) was significantly decreased compared with the allograft group (131.01 ± 5.69 *μ*m; *P* < 0.05). Furthermore, the Ki-67 expression (Figures 1(b1)–1(b3)) in VSMCs was also significantly decreased in the lycopene group compared with that in the allograft group. This indicated that VSMCs proliferation was reduced in grafted vessels after lycopene treatment. However, there was no obvious improvement in the caspase-3 expression (Figures 1(c1)–1(c3)), which indicated that the proapoptosis effects of lycopene were not significant in this study. The ratio of Ki-67/caspase-3 positive cells was analysed to evaluate the proliferation and apoptosis levels of the intimal cells. The ratio in the lycopene group (3.41 ± 0.21) was significantly lower than in the allograft group (7.16 ± 0.29).

### 3.3. Downregulation of ROCK1 and ROCK2 Expression Levels in Allograft Vessels after Lycopene Treatment

**Figure 2:**
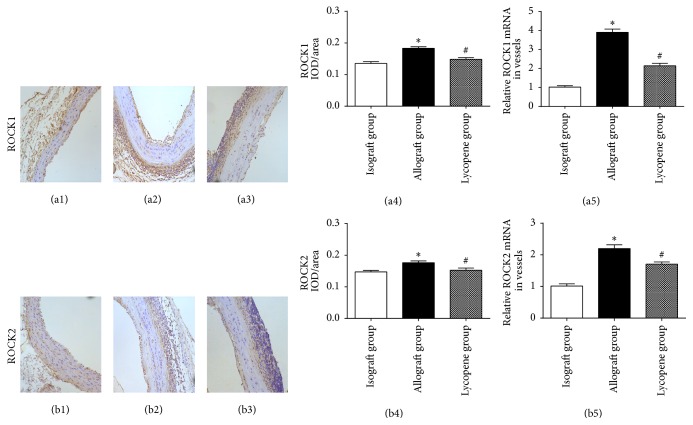
Expression of ROCK1 and ROCK2 in each group. ((a1)–(a3) and (b1)–(b3)) Immunohistochemical staining of ROCK1 and ROCK2 in grafted arteries (original magnification ×200). ((a4) and (b4)) The mean of the IOD/area of ROCK1 and ROCK2 in each group. IOD/area: integrated optical density/area; the mean of the IOD/area of positive staining was used to accurately measure the degree of positive expression in immunohistochemical staining. ^*∗*^ *P* < 0.01 for the isograft group compared with the allograft group; ^#^ *P* < 0.05 for the lycopene group compared with the allograft group. ((a5) and (b5)) Real-time quantitative PCR analysis of ROCK1 and ROCK2 mRNA expression in each group. ^*∗*^ *P* < 0.001 and ^#^ *P* < 0.05. ((a1) and (b1)) Isograft group; ((a2) and (b2)) allograft group; ((a3) and (b3)) lycopene group.

ROCKs activation was also critical in the progression of vascular arteriosclerosis. The ROCK1 and ROCK2 immunohistochemical staining was performed in our study to demonstrate the link between the upregulated ROCKs expression and arteriosclerosis. As shown in Figures 2(a1)–2(a3) and 2(b1)–2(b3), the ROCK1 and ROCK2 expression levels in VSMCs were significantly decreased in the lycopene group ((a3) and (b3)) compared with the allograft group ((a2) and (b2)). The decreased IOD/area means (integrated optical density/area) of ROCK1 and ROCK2 in the lycopene group ((a4) and (b4)) coincided with the immunohistochemical staining results. Real-time quantitative PCR analysis also revealed that the relative mRNA expression of ROCK1 and ROCK2 ((a5) and (b5)) was significantly lower than that in the allograft group (both *P* values < 0.05).

### 3.4. Lycopene Inhibited ICAM-1 Expression in Allograft Vessels

**Figure 3:**
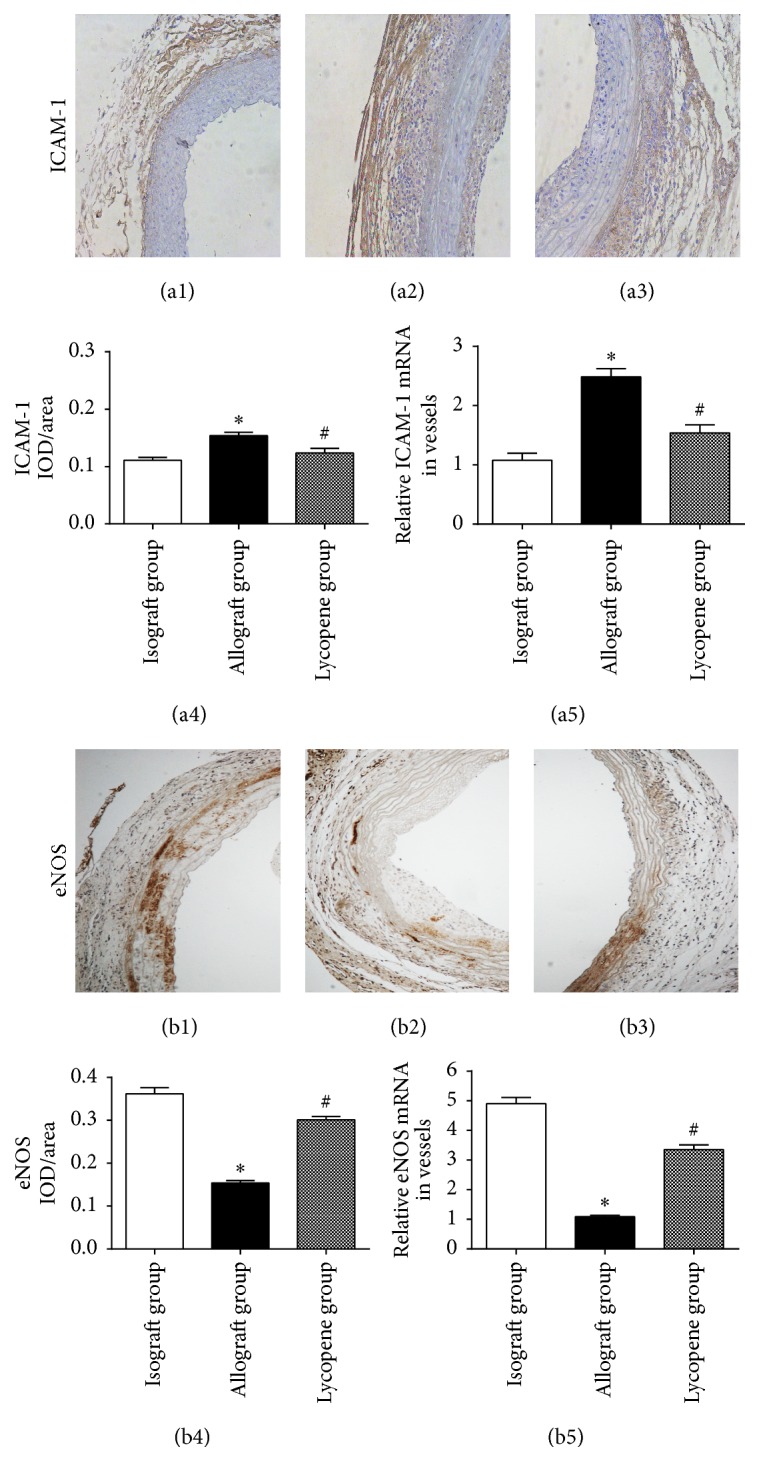
Expression levels of ICAM-1 and eNOS were determined by immunohistochemical staining and real-time quantitative PCR analysis. ((a1)–(a3) and (b1)–(b3)) Immunohistochemical staining of ICAM-1 and eNOS in grafted arteries (original magnification ×200). ((a4) and (b4)) The mean of IOD/area of ICAM-1 and eNOS immunohistochemical staining in each group. (a4) ^*∗*^ *P* = 0.001, allograft group compared with the isograft group; ^#^ *P* = 0.019, lycopene group compared with the allograft group. (b4) ^*∗*^ *P* < 0.001, ^#^ *P* < 0.001. ((a5) and (b5)) Real-time quantitative PCR analysis of ICAM-1 and eNOS mRNA expression in each group. (a5) ^*∗*^ *P* < 0.001, ^#^ *P* = 0.003. (b5) ^*∗*^ *P* < 0.001 and ^#^ *P* < 0.001. ((a1) and (b1)) Isograft group; ((a2) and (b2)) allograft group; ((a3) and (b3)) lycopene group.

We assessed whether lycopene inhibited inflammation in the grafted vessels, which was also considered to play a key role in the progress of allograft arteriosclerosis. In this study, immunohistochemical staining of grafted vessels was performed, and the results are shown as the IOD/area means (Figure 3(a4)). As shown in Figure 3, there is a significant decrease in the ICAM-1 expression in the lycopene group (a3) compared with the allograft group vessels (a2). The real-time quantitative PCR results (a5) also demonstrated that the relative mRNA expression of ICAM-1 in the lycopene group was significantly lower than that in the allograft group.

### 3.5. Lycopene Increased eNOS and PKG-I Protein Expression in Aortic Allografts

**Figure 4:**
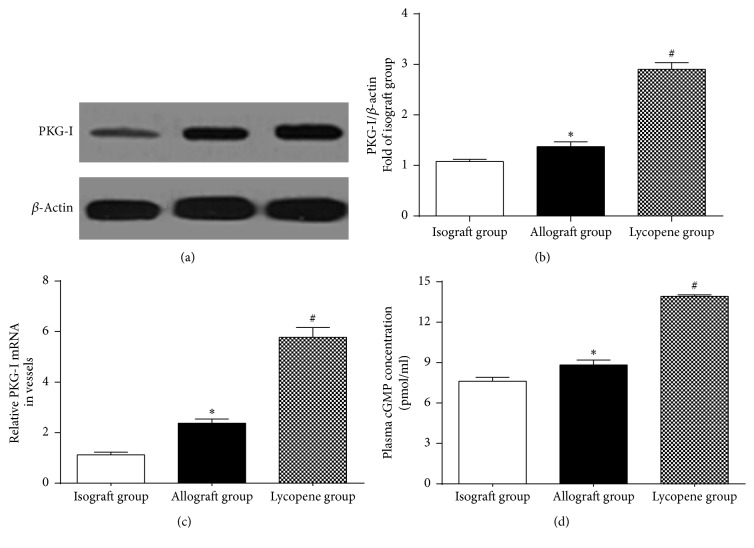
Analysis of PKG-I protein expression in grafted arteries and plasma cGMP concentration detection in each group. ((a) and (b)) Western blot analysis of PKG-I protein expression in each group; ^*∗*^ *P* < 0.05, allograft group compared with the isograft group; ^#^ *P* < 0.001, lycopene group compared with the allograft group. (c) Real-time quantitative PCR analysis of PKG-I mRNA expression; ^*∗*^ *P* < 0.001; ^#^ *P* < 0.001. (d) Determination and analysis of the plasma cGMP concentration in each group; ^*∗*^ *P* < 0.05; ^#^ *P* < 0.001.

eNOS is an important factor which has a protective function in the cardiovascular system and is attributed to the production of NO in vascular endothelial cells. In this study, eNOS expression level was measured by immunohistochemical staining of grafted vessels in each group (Figures 3(b1)–3(b3)). The positive staining was evident in the isograft group (b1) and lycopene group (b3), while, in the allograft group (b2), it was significantly decreased. The IOD/area means analysis (Figure 3(b4)) and real-time quantitative PCR measurements (b5) both demonstrated the results of the immunohistochemical staining. All these data revealed that eNOS expression level in arterial transplantation could be increased after lycopene treatment. Meanwhile, PKG-I plays a critical role in regulating vascular smooth cells by regulating Rho A activation. Western blot analysis was performed to evaluate the protein expression of PKG-I in grafted vessels. As shown in Figure 4(a), the results of Western blot analysis revealed the different expression of PKG-I protein in aortic grafts between the 3 groups. In the lycopene group, the PKG-I expression was markedly increased compared with the allograft group (*P* < 0.05). The result also coincided with the real-time quantitative PCR analysis of PKG-I mRNA expression in the grafted vessels (Figure 4(b)).

## 4. Discussion

Transplant vasculopathy is one of the major causes of graft failure in organ transplantation. The protection of transplant vessels has become an important issue in maintaining the normal functions of the grafts [13, 14]. In this study, we investigated the preventive and therapeutic effects of lycopene in treating transplant vasculopathy. The results of our study demonstrated that intimal hyperplasia and smooth muscle cell proliferation were remarkably reduced by the administration of lycopene at 8 weeks after allogeneic aortic transplantation. Furthermore, we also found that lycopene could increase the expression of PKG-I and eNOS, downregulate the expression of ROCK1, ROCK2, and ICAM-1, and inhibit the infiltration of inflammatory cell in allograft vessels. However, in the vascular intima and media, the immunohistochemical staining showed that there was no significant increase in the cell apoptosis after lycopene treatment. This indicated that the proapoptosis effect of lycopene was not obvious in this study, and its ameliorative effects on the transplant arteriosclerosis were not based on the proapoptosis mechanisms. Furthermore, the plasma cGMP concentration was also detected in this study to evaluate the expression levels of cGMP signaling pathways. After lycopene treatment, it exhibited a significant increase when compared with the allograft group. All the data in this study demonstrated that arteriosclerosis in vascular allograft transplantation could be ameliorated by lycopene through regulating NO/cGMP pathways and Rho-associated kinases expression.

Endothelial cells are extremely important in regulating vascular tone and maintaining vascular structure [15]. However, the immune and nonimmune injuries that chronically occur in endothelial cells (ECs) may trigger endothelial activation and dysfunction in patients after organ transplantation [16]. These can result in intimal hyperplasia, smooth muscle cell (SMC) proliferation, and extracellular matrix synthesis, which is accompanied by resulting gradual decline in graft function. Nitric oxide (NO) is the primary mediator of endothelial function, and it can cause vasodilation by upregulating guanylyl cyclases on subjacent vascular SMC and inhibiting vascular SMC proliferation and migration [17, 18]. However, ischemia-reperfusion (I/R) injury, oxidative stress, and immunosuppressive drugs can generate excessive O_2_^−^, which can rapidly inactivate NO in allografts and lead to the occurrence and progression of graft vasculopathy [19]. In this study, the eNOS immunohistochemical staining was performed in grafted vessels to analyse the expression levels of NO* in vivo*. eNOS has a protective effect on the cardiovascular system and is considered to be attributed to NO production in the vascular endothelial cell. Its activation and expression play important roles in regulating the NO production. Lycopene is the most potent single oxygen quencher among the natural carotenoids. Di Tomo et al. demonstrated that lycopene suppresses reactive oxygen species (ROS) generation and increases the NO expression level and bioavailability in human umbilical vein endothelial cells [6]. The authors also reported that lycopene regulates the NO expression level and bioavailability by directly reducing molecules. The results of our study showed that neointimal hyperplasia was alleviated, and the Ki-67 expression was downregulated in the lycopene group. In addition, these results coincided with the experimental design proposed in this study that lycopene can improve the graft vasculopathy by antioxygenation. Cells apoptosis dysfunction of vascular smooth muscle cell is considered another important factor that causes neointimal hyperplasia and intimal thickening during the progression of vascular atherosclerosis [20]. The proapoptosis effect of lycopene was not obvious in our study which indicated that lycopene ameliorated vascular atherosclerosis through regulating other signaling pathways, and this is in agreement with our experimental hypothesis.

Migration of vascular smooth muscle cell from the media to intima plays a crucial role during the formation of atherosclerotic plaques and vascular restenosis in graft vasculopathy. Rho-associated kinases (ROCKs) are typical downstream effectors of the small GTPase Rho family [21]. ROCKs can be activated by upstream regulator Rho A, phosphorylating and inactivating myosin light chain (MLC) phosphatase, and subsequently enhancing MLC phosphorylation, resulting in vasoconstriction [22, 23]. Additionally, ROCKs can regulate the actin cytoskeleton reorganization of endothelial cells (ECs) and SMC, which is associated with vascular remodeling [9]. In a porcine model, long-term inhibition of Rho kinase results in regression of the arteriosclerotic lesions [24]. ROCK1 and ROCK2 are two representative subtypes of the ROCK family. They have high homology, and 65 percent of the amino acid sequences are the same. In addition, both help regulate many important basic functions of cells, such as cell adhesion, migration, proliferation, apoptosis, and transcription regulation. The upregulated ROCK1 and ROCK2 expression levels are considered to play an important role in the progression of transplant vascular atherosclerosis [24]. The results of our study also demonstrated that ROCK1 and ROCK2 expression levels were significantly decreased after lycopene treatment, which improved the grafted vessels. Piera-Velazquez et al. showed that ROCK1 inhibition can decrease foam cell formation and inhibit the pathological process of atherosclerosis [25]. However, the specific regulatory mechanisms of ROCK1 and ROCK2 in ameliorating graft atherosclerosis remain unclear. In addition, PKG-I is a critical mediator of the NO/cGMP pathway that plays an important role in regulating vascular smooth cell by regulating Rho A activation [26]. PKG-I phosphorylates Rho A at Ser188, which inhibits its membrane association and prevents the activation of Rho kinases [27]. Our data showed that lycopene could increase the PKG-I level and downregulate ROCK1 and ROCK2 expression in grafted vessels. The increased plasma cGMP concentration also indicated the upregulated expression of cGMP signaling pathway after lycopene treatment.

Vessel inflammation is also an early and important event in the process of allograft atherosclerosis [28]. It is now widely accepted that both the production of intercellular adhesion molecules (ICAM-1) and their shedding onto endothelial and leukocytic surfaces play a pivotal role in mediating the interaction between endothelial cells and blood constituents at the early stage of atherosclerosis [29, 30]. In addition, ICAM-1 increased the thrombotic potential by influencing the migration and proliferation of SMCs in the vessel blood. In addition, it is reported that TNF-*α* can upregulate ICAM-1 expression on the endothelium during atherogenesis via NF-*κ*B binding to the promoter. It has been reported that lycopene can inhibit TNF-*α*-induced ICAM-1 protein expression through affecting the NF-*κ*B signaling pathway [31], which explains how lycopene has protective effects on allograft atherosclerosis through anti-inflammatory activity.

In conclusion, the results of our study demonstrated that lycopene had significant therapeutic effects on the occurrence and progression of transplant vascular atherosclerosis. It could ameliorate allograft atherosclerosis by regulating the expression of the NO/cGMP pathway and affecting many critical factors. This may provide new possibilities for preventing the formation of grafts atherosclerosis during clinical organ transplantation.
